# Supplementary material for: BCL2 Protein Progressively Declines during Robust CLL Clonal Expansion: Potential Impact on Venetoclax Clinical Efficacy and Insights on Mechanism
Source: Lymphatics. Author manuscript; Available in PMC 2024 Dec 11. (PMC11632909; doi:10.3390/lymphatics2020005)
Supplement: Supplementary [file NIHMS2026894-supplement-Supplementary.zip › lymphatics-2788756-supplementary.pdf]

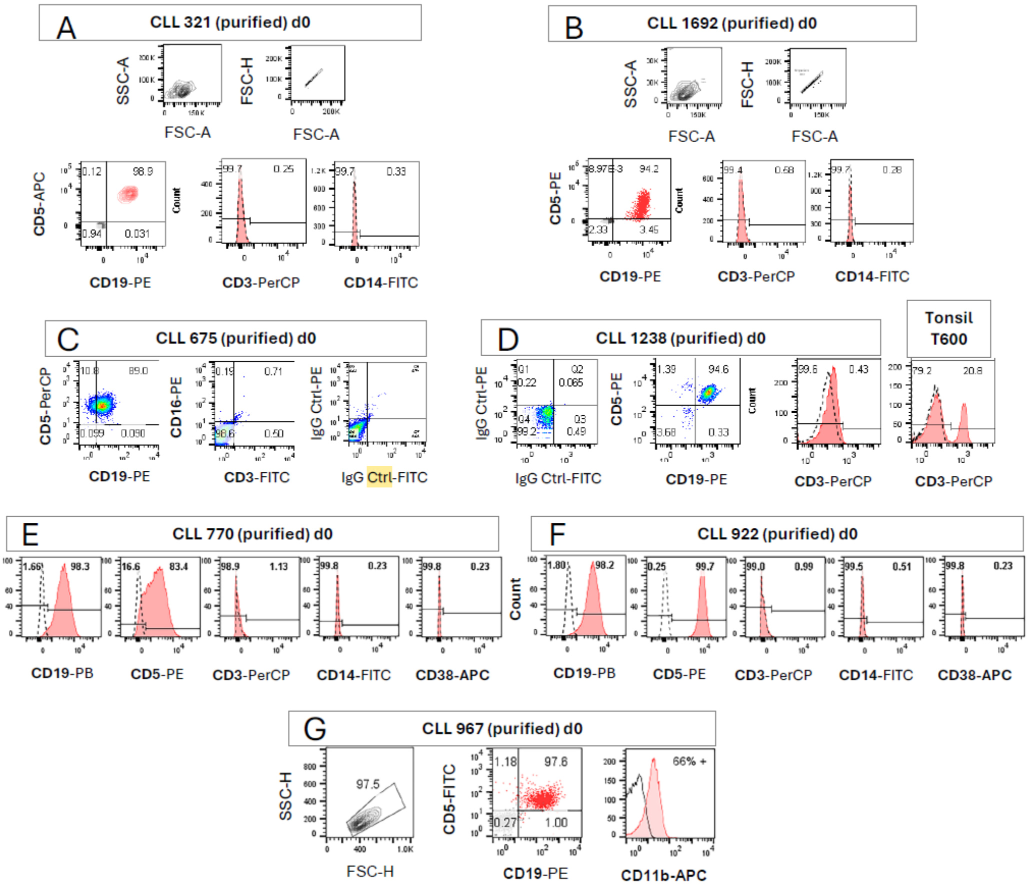

Supplementary Figure S1. Surface marker characterization of purified CLL populations *prior* to culture. Purified CLL preparations were subjected to reanalysis for surface markers following recovery from frozen aliquots at d0. (**B-D and G**) populations were uniformly CD19+/CD5+ with variations in the intensity of CD5 and CD19 staining that may reflect differing fluorophores employed as well as intrinsic differences between clones (such variations have been reported between diverse CLL clones for CD19 (doi.org/10.1002/cyto.b.20030) and similarly within clones for CD5 (doi: 10.1016/j.exphem.2016.09.010; doi.org/10.2119/molmed.2011.00360). (**E-F**) CLL770 and CLL922 were not co-stained for CD5/CD19 but were highly positive for each when independent. (**A**) **CLL321** was determined to be 99% CD19+/CD5+ with <1% CD3+ and CD14+ contaminants. (**B**) **CLL1692** was 98% CD19+, with 94% of these exhibiting CD5 above the set threshold and <1% CD3+ and CD14+ contaminants. (**C**) **CLL675** was 89% CD19+/CD5+, with 11% expressing CD5 but not CD19 (just below the set threshold). Because the CD5 intensity of the latter was equivalent to that of cells co-staining with CD5+/CD19+ and because CD3+ cells were <1%, we conclude these represent CLL cells and not contaminating T cells. Contaminating NK cells were not evident (< 0.2% CD16+). (**D**) CLL1238 was 95% CD19+/CD5+ with 0.43% CD3+ T cells. (**E-F**) CLL770 & CLL922 cells were uniformly positive for CD19 and for CD5 (CLL922 > CLL770 for CD5) and negative for CD3, CD14, and CD38 (found on NK cells as well as activated T and B cells and memory B cells). (**G**) CLL967 was 98% CD19+/CD5+, with the majority of the latter co-expressing CD11b (expressed on some CLL clones and linked to clinical aggressiveness (doi.org/10.1371/journal.pone.0254853).

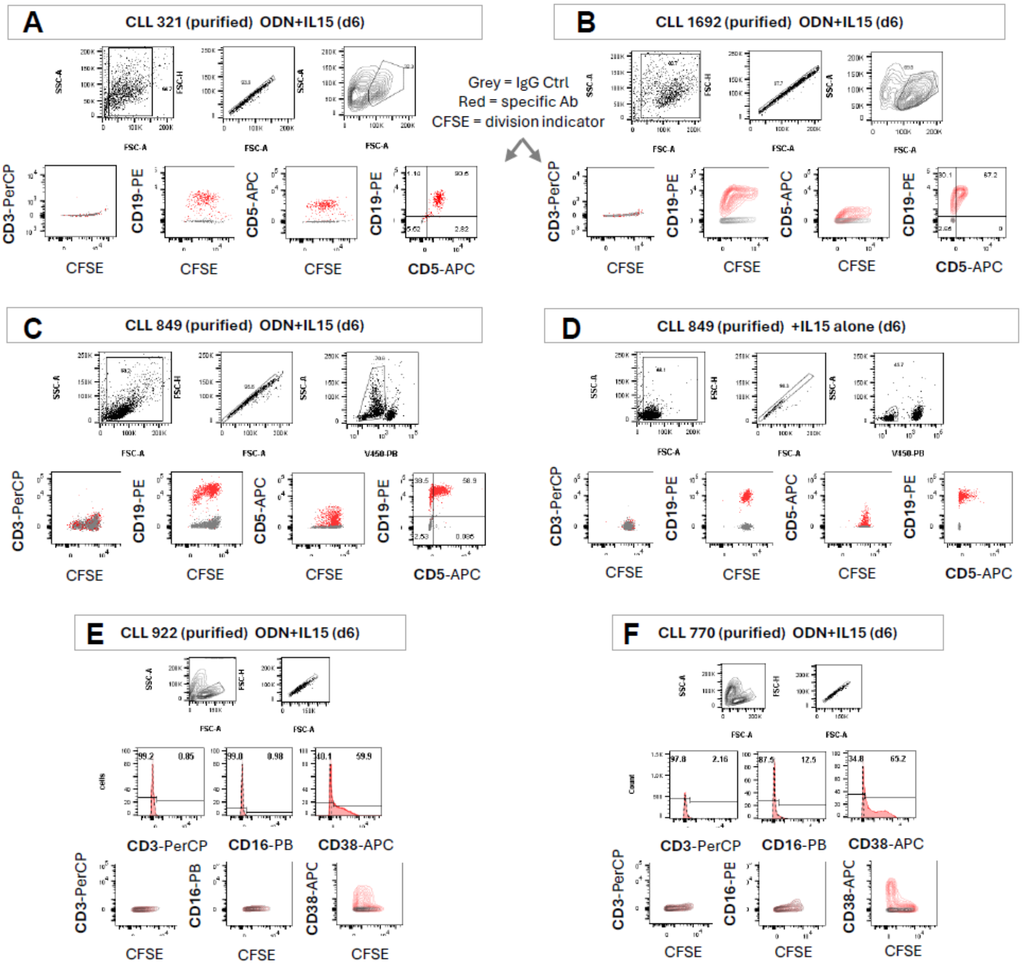

Supplementary Figure S2. CD19+/CD5+ CLL phenotype assessed within ODN+IL15-induced cycling cultures of purified CLL cell populations. (**A**) U-CLL321, (**B**) U-CLL1692 (**B**), (**C-D**) M-CLL849, (**E**) M-CLL922, and (**F**) U-CLL770 were each labeled with division-tracking dye, CFSE, and stimulated for 6 d with ODN+IL15 (**A-C, E-F**) or IL-15 alone (**D**). Harvested cells were stained with fluorochrome-conjugated specific mAbs or IgG controls. By flow cytometry, events were gated to remove debris, dead cells, and cell aggregates and then analyzed for fluorescence. In the above plots, "red" events represent cells stained with specific Abs; "grey" events represent cells stained with control Abs. Plots of Ab fluorescence (log) as a function of CFSE fluorescence (log) unequivocally show that cycling cells exhibit a CD19/CD5+ CLL phenotype but lack the T cell marker CD3. Of note, while cycling CLL321 cells (**A**) retain equivalent CD5 and CD19 levels as divisions progress, in the case of CLL-1692 (**B**) and CLL-849 (**C**) (two CLL with relatively low CD5 expression when quiescent), the extensively divided cells showed reduced levels of CD19 and likely CD5. This might reflect CLL differentiation to a plasmablast phenotype as seen in CLL cultures activated by ODN+CD40L+cytokines (doi.org/10.3389/fimmu.2014.00292) and for CD19 when normal B cells transition from plasmablasts to plasma cells (doi: 10.4049/jimmunol.1501761). Alternatively, the decline reflects the transformation to Richter-type cells. In a proportion (~30%) of CLL to Richter's transformations, both CD5 and CD19 levels declined from those expressed in the original CLL population (doi.org/10.1002/hon.3165_495). Importantly, CD3+ T cells were not detectable in 5/5 of the ODN+IL15-activated day 6 cultures (**A-F**). Furthermore, in 2/2 such populations tested for CD16+ NK cells (**E, F**), none were detected. Together, these assessments indicate that the B cell purification step for depleting non-B cells from the CLL population does effectively remove possible contaminating CD8+ T cells or NK cells that could mount a vigorous proliferative response to IL15 (doi.org/10.1084/jem.20020772; doi.org/10.1084/jem.180.4.1395).

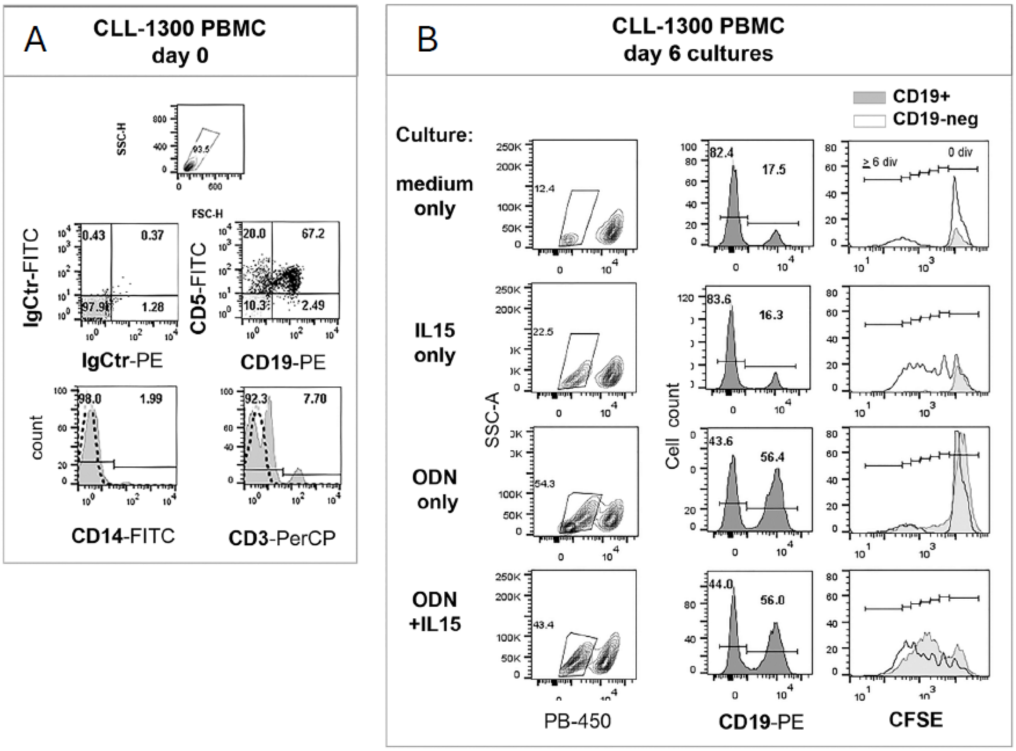

Supplementary Figure S3. CLL1300 PBMC surface marker expression at t=0 and after 6 d culture with ODN+IL15. CLL1300 was the single CLL preparation used as PBMC that did not undergo a negative depletion step for B cell purification. Combined findings from surface marker staining (**A**) prior to culture (**B**) after 6 d activation (left staining columns for surface staining and CD19 gating) show that non-B cell contamination in the original PBMC (in A, indicated by the presence of 8% CD3+ T cells) is linked to the significant clonal expansion of CD19-negative cells (B right column; CFSE plots). Of note, the CD19-negative subset proliferated extensively, both in cultures with IL15 alone or ODN+IL15 (only a small subset of the latter proliferated in the medium alone). In agreement with our past findings with purified CLL populations, the CLL1300 CD19+ compartment showed no cycling in cultures with medium alone or IL15 alone; it showed only minimal division in ODN-only cultures but manifested notable cycling in cultures stimulated with both ODN+IL15. Initial contamination with CD8+ T cells and NK cells in isolated PBMC likely explains the unusually significant proliferation in IL15-only cultures (never observed when using purified CLL preparations) because both CD8+ cytotoxic T cells and NK cells can replicate extensively with IL15 alone (doi.org/10.1084/jem.20020772; doi.org/10.1084/jem.180.4.1395). Of note, the contaminating CD19-negative cells represented the majority of progeny with the greatest cycling history (4 to >6 divisions), while CD19+ cells dominated (64%) in cells manifesting 0 to 3 divisions. Based on CD19+ cell dominance in the latter, when data from CLL1300 are included in the study, only cells with 0 to 3 divisions are assessed.

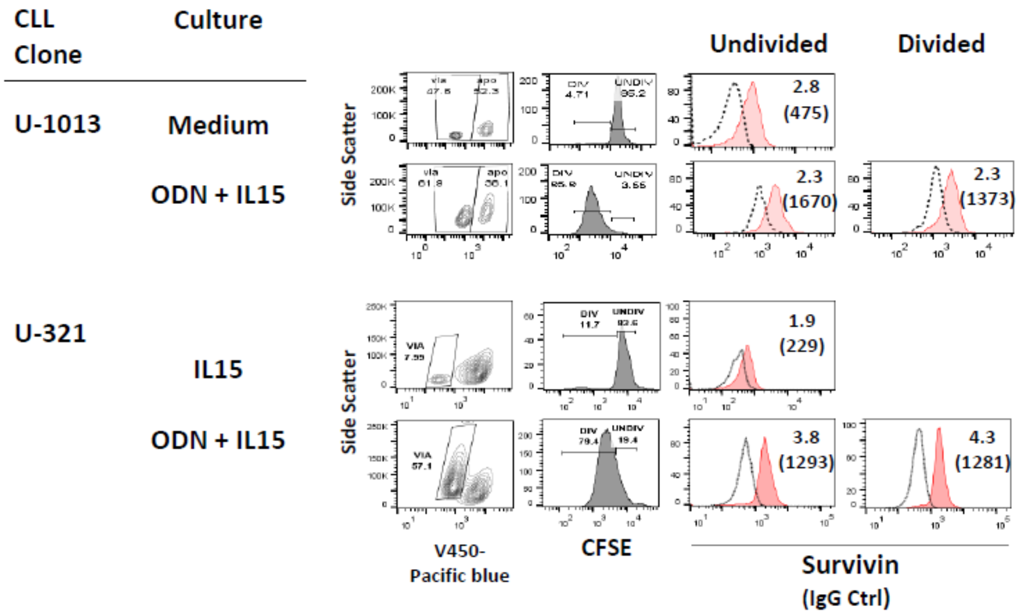

Supplementary Figure S4. Survivin protein in cultured B-CLL cells. CFSE-labeled U-1013 and U-321 cells were cultured for 4 or 5 days, respectively, with the indicated stimuli prior to exposure to viability-excluding dye, fixation, and staining for cytoplasmic survivin protein (Methods and Materials). Viability-gated cells were then gated as divided and undivided, based on CFSE fluorescence and gated subsets analyzed for PE fluorescence, with anti-survivin staining = filled histograms; IgG control = unfilled). Inserted values represent RMFI (and in parenthesis, Δ MFI). Detection of survivin within non-proliferating B-CLL cells cultured with IL-15 alone (e.g., U-1013) was unexpected but is consistent with occasional reports of survivin in freshly isolated B-CLL from blood (doi.org/10.1182/blood.V97.9.2777). Of potential relevance, a recent study with cycling mouse B cells found that, in contrast to absent survivin in B cells that enter G1 from a resting state (G_0), this protein is present and functional in B cells that re-entered G1 following recent extensive cycling (doi.org/10.1073/pnas.2115567119). Thus, baseline survivin might reflect the past cycling history of B-CLL cells isolated from blood. In contrast to survivin+ mouse B cells in G1 that could undergo mitogen-independent cycling (doi.org/10.1073/pnas.2115567119), this was not observed with U-CLL1013 cells (above and in our past study doi.org/10.4049/jimmunol.1403189). That said, the U-1013 population was atypical in its more accelerated cycling within ODN+IL15-stimulated cultures (data not shown).

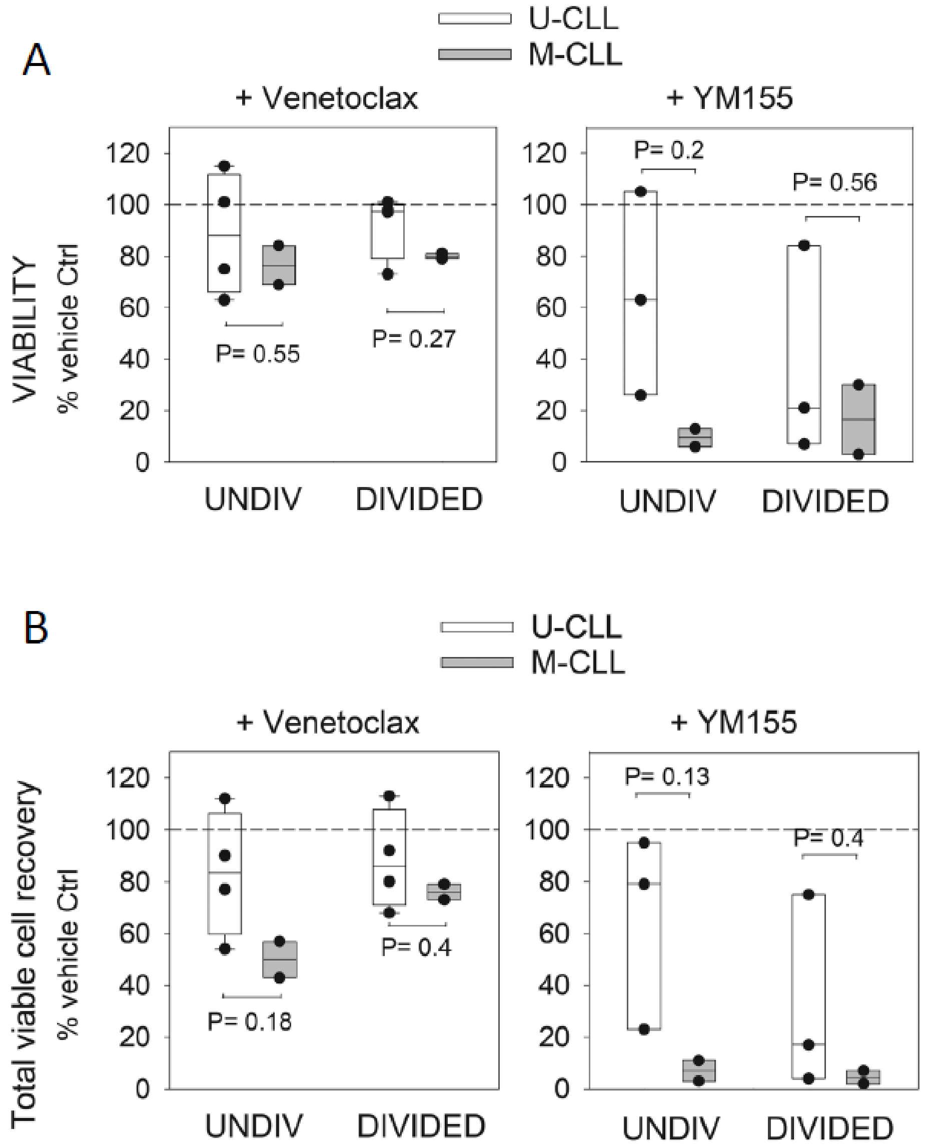

Supplementary Figure S5. Comparison of ODN+IL15-stimulated U-CLL versus M-CLL cells following treatment with venetoclax or YM155. CFSE-labeled CLL cells were stimulated on d0 and pulsed with limiting doses of either venetoclax (17 nM) or YM155 (333 nM) or vehicle alone at 24-36 h prior to culture harvest at d5(d6) for determining (**A**) viability or (**B**) absolute viable cell yield. For venetoclax experiments, the following were tested: U-CLL: 321, 675, 996, and 1013 and M-CLL: 275 and 1031. YM155 susceptibility experiments involved the following: U-CLL: 321, 1013, and 1692 and M-CLL: 849 and 1031. P values are determined by a 2-sided, unpaired t-test. While not reaching statistical significance, there is a suggestion that U-CLL cells (undivided and divided) might be more resistant than M-CLL cells to both venetoclax and YM155-induced death at limiting inhibitor doses.
